# A Comprehensive Study on the Mechanical Properties of Different 3D Woven Carbon Fiber-Epoxy Composites

**DOI:** 10.3390/ma13122765

**Published:** 2020-06-18

**Authors:** Qiaole Hu, Hafeezullah Memon, Yiping Qiu, Wanshuang Liu, Yi Wei

**Affiliations:** 1 School of Textile and Garment, Anhui Polytechnic University, Wuhu 41000, China; huqiaole@ahpu.edu.cn; 2 Key Laboratory of Textile Science &Technology, Ministry of Education, College of Textiles, Donghua University, 2999 North Renmin Road, Shanghai 201620, China; hm@mail.dhu.edu.cn (H.M.); ypqiu@dhu.edu.cn (Y.Q.); 3 Donghua University Center for Civil Aviation Composites, Donghua University, 2999 North Renmin Road, Shanghai 201620, China; 4 College of Textiles and Apparel, Quanzhou Normal University, Quanzhou 362000, China

**Keywords:** 3D woven composite, carbon fibers, through-thickness properties, mechanical properties

## Abstract

In this work, the tensile, compressive, and flexural properties of three types of 3D woven composites were studied in three directions. To make an accurate comparison, three 3D woven composites are made to have the same fiber volume content by controlling the weaving parameters of 3D fabric. The results show that the 3D orthogonal woven composite (3DOWC) has better overall mechanical properties than those of the 3D shallow straight-joint woven composite (3DSSWC) and 3D shallow bend-joint woven composite (3DSBWC) in the warp direction, including tension, compression, and flexural strength. Interestingly their mechanical properties in the weft direction are about the same. In the through-thickness direction, however, the tensile and flexural strength of 3DOWC is about the same as 3DSBW, both higher than that of 3DSSWC. The compressive strength, on the other hand, is mainly dependent on the number of weft yarns in the through-thickness direction.

## 1. Introduction 

Carbon fiber reinforced polymer composites have been used successfully in aircraft, rails, automotive, water vehicles, and sports devices [1]. In these applications, 2D laminates were used primarily due to their outstanding in-plane performance, higher stiffness-or strength-to-weight ratio, and fabrication convenience. Because of the lacking of reinforcements in the through-thickness direction, the out-plane mechanical properties are resin dominated and often lower than those of in-plane, limiting their use in many structural applications where complex loadings are present. Comparatively, composites made of 3D woven fabrics have higher z-direction strength, delamination resistance, and damage tolerance, together with better integrity and design flexibility [2,3]. Furthermore, the 3D woven preforms can be made conveniently on standard industrial weaving looms (i.e., a rapier loom) by minor machine modifications (i.e., by increasing the number of heald frames and/or rapier picks) [4,5]. 

It is known that the geometric arrangement of fibers has a great influence on the properties of composite materials [6,7,8,9]. For 3D woven fabrics, various complex structures are prepared through changing yarn interlace patterns, which makes the analytical or numerical prediction of their mechanical behavior significantly more complicated than usual 2D laminate [10,11]. As a result, there is an apparent need to obtain experimental data from which theoretical models can be built to evaluate and predict important mechanical properties so as to serve as a guide for structural selection and design.

Unfortunately, experimental data for 3D composites are limited, particularly in the case of 3D carbon woven fabric reinforced composites. The majority of the existing experimental data of 3D composites are based on the effect of weaving parameters [12,13], in-plane tension [14,15,16], and compression [17,18,19,20]. Very few articles reported data based on different structures with the same weaving parameters settings and with the same fiber volumes for the in-plane properties of 3D composites [8,21,22,23,24,25,26,27]. Data on through-thickness (z-direction) properties are even harder to find, mainly because of the difficulty to directly measure out-plane mechanical properties while keeping the composites have the same weaving parameters and fiber volumes. Therefore, a more in-depth study is needed in order to understand how the fabric architectures affect the overall mechanical behavior of composites so that optimal 3D structures can be designed and utilized. 

In this study, three typical woven architectures were selected, 3D orthogonal (3DOW), 3D shallow straight-joint (3DSSW), and 3D shallow bend-joint (3DSBW). The weaving parameters setting and fiber volume of these structures were carefully and accurately controlled and were kept consistent with another. A Vacuum-Assisted Resin Infusion (VARI) process was used to fabricate the 3D composites. Properties in the x, y and z-directions (i.e., warp, weft, through-thickness direction) of these composites were tested and compared with each other to determine which one has the overall better mechanical performance.

## 2. Materials and Methods

### 2.1. Materials

The carbon fibers used in this study were a T800 grade 12K (SYT55S-12K) carbon fibers provided by Zhongfu Shenying Carbon Fiber Co., Ltd. Lianyungang, China. The three types of woven fabrics were manufactured by Huaheng High Performance Fiber Textile Co., Ltd. Yixing, China. The schematics of the three woven fabrics are shown in Figure S1. The matrix was an epoxy resin, BAC 172, supplied by Zhejiang Baihe Advanced Composites Ltd. Hangzhou, China. The details of the raw materials (carbon fiber and resin) used in this study are listed in Tables S1 and S2.

### 2.2. Fabrication of the Composites

The self-explanatory diagram of the composite fabrication process via VARI is illustrated in Figure S2. In this process, resin injection temperature was 24 ± 2 ℃, and the curing condition was 1 h at 120 ℃ in the oven with the heating rate of 2 ℃/min and the pressure in the whole process was kept at 0.098 MPa by a vacuum pump (Welch, MPC301Z, Shanghai, China). The final dimension of the composites obtained was 200 mm length, 200 mm width and 14.5 mm thick, and the average fiber volume fraction was approximately 45%, calculated by the weight method.

### 2.3. Characterization of the Mechanical Properties 

The mechanical properties of the three woven composites were tested on a computer-controlled electronic universal testing bench LD26-5105 (Labscans, Shenzhen, China) with different load cell (i.e., the load cell capacity for out-plane tension was 50 KN), while the other tests (i.e., in-plane and out-plane compression and flexural, in-plane tension) were done with the 100 KN load cell capacity. It is noteworthy that the thickness of the test coupons must exceed the unit cell thickness $\;t_{unit}$, which were measured by the fluorescence microscope (FM-400C, Pudan, Shanghai, China) and the measured results were listed in Table S3. One exception is the 3DOWC due to the presence of the through-thickness z-binder yarn. Considering the z-yarn is theoretically vertical in the thickness direction, their contribution to warp/X and weft/Y direction is negligible in this study.

The preparation of testing coupons was scaled down in accordance with the ASTM D4762 [28] and their detailed information was shown in Figures S3–S5. For in-plane tensile and compression tests, the stiffener tabs, 1.5 mm, made out of [45/-45]_2s_ glass fiber laminates were used at both ends of the test coupons. Because of the short length at the z-direction, I-shape tensile test coupons were machined (Coupon 3) for out-plane tensile test according to the previous research [29,30]. The crosshead speed for the tensile and compression tests was 2.0 mm/min and 1 mm/min, respectively. The strain was measured by the strain gauge. The crosshead speed for the flexural test was 1.0 mm/min. For each direction test, five coupons were tested.

In addition, it was worth noting that the stress shown in the stress-strain curve was not the final stress, due to the fact the strain gauge could easily fail before the final failure occurred.

## 3. Results and Discussion

### 3.1. Fiber Volume Fraction

**Figure 1:**
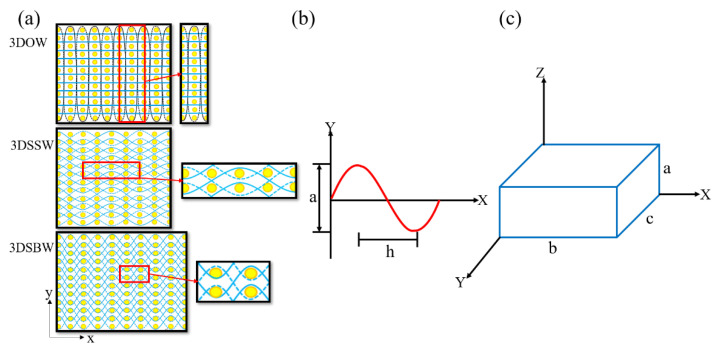
(**a**) Unit cells of 3D fabrics; (**b**) the warp or z-yarn path; (**c**) the simplified model of the unit cell.

In order to accurately measure and properly compare the properties of the composites made of the 3D fabrics, keeping the fiber volume consistent is of particular importance. In this study, unit cells of the representative structures, shown in Figure 1a, were selected and simplified into a rectangle model, as shown in Figure 1c to calculate the weaving parameters setting. For the convenience of analysis, for 3DSSW and 3DSBW, the warp yarn path was approximated as a sine curve, as shown in Figure 1b, and the weft yarn was considered as a straight line. While for 3DOW, the z-yarn was regarded as a sine curve and the warp and weft yarns were treated as straight lines. So, the warp yarn (z-yarn) path Y and length L are defined as follows:

(1) Y=(a/2)sin(πx/h)

(2) $$L=2\overset{h}{\underset{0}{\int }}\sqrt{1+{\left(Y^{\prime }\right)}^{2}}dx=2\overset{h}{\underset{0}{\int }}\sqrt{1+{\left(\pi a/\left(2h\right)\right)}^{2}cos^{2}\left(\pi x/h\right)}$$

The yarn cross-sectional area,$\;S_{yarn}$ and the dimension of the model shown in Figure 1c can be calculated by Equations (3) and (4):

(3) $$S_{yarn}=N_{tex}/\rho$$

(4) $$a=T/n;\;\;b=2h=\frac{20}{P_{w}};\;\;\;c=\frac{20}{P_{j}\;}$$

Base on Equations (5)–(7), the fiber volume $V_{f}\;$is defined for the three type of 3D structures as follows:

(5) $$3\mathrm{DOW}:\;V_{f}=(V_{x}+V_{y}+V_{z})/V_{unit}=(N_{x}\times 2h+N_{y}\times 4c+4L)\times S_{yarn}/\left(a\times b\times c\right)\;$$

(6) $$3\mathrm{DSSW}:V_{f}=(V_{x}+V_{y})/V_{unit}=\left(4L+8c\right)\times S_{yarn}/\left(a\times b\times c\right)$$

(7) $$3\mathrm{DSBW}:V_{f}=(V_{x}+V_{y})/V_{unit}=\left(4L+4c\right)\times S_{yarn}/\left(a\times b\times c\right)$$

where,

*a* is the thickness of the unit cell in a woven fabric;

*b* and *c* are the distance between the weft and warp yarn with the same interlace way, respectively;

*n* is the number of unit cells in the through-thickness direction of the fabric;

T is the thickness of the woven fabric;

*h* is the distance between two adjacent weft yarn in the unit cell;

*ρ* is the density of the woven yarn;

$N_{tex},N_{x},N_{y}\;are\;$the yarn density, the warp and weft yarn layer of the 3D fabric, respectively;

$P_{j},P_{w}$ are the warp and weft yarn densities of the 3D fabric, respectively;

$V_{x},V_{y,}V_{z},V_{unit}$ are the fiber volumes in x-, y- and z-directions and the unit cell, respectively.

From Equations (5)–(7), the fiber volume was determined by warp and weft yarn density, together with the number of layers of the fabric as well as the fabric thickness. Thus, in order to obtain the same weaving parameters setting, the target fiber volume (45%), and the warp yarn target density (10 picks/cm), the warp layers and the fabric thickness need to be set as 21 layers and 16 mm, respectively. After inputting the number of layers and fabric thickness into Equations (3)–(7) using Mathematica (a software program, Mathematica 12.0, Wolfram Research, Champaign County, IL, American), the weft yarn density could be calculated. These calculated parameters were subsequently used as the starting set points on the weaving machine. The weaving parameters setting and fiber volumes that were actually obtained were displayed in Table 1. 

### 3.2. Morphology of Woven Fabrics 

**Figure 2:**
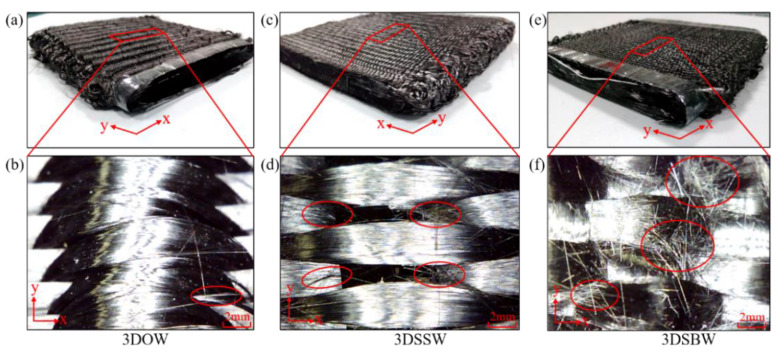
The morphology of three woven fabrics (**a**,**b**) 3DOW, (**c**,**d**) 3DSSW, (**e**,**f**) 3DSSW.

The observation of fabric morphology is necessary to understand the degree of fiber fracture under the same weaving parameters setting. The most serious fiber fracture, as shown in Figure 2f, was seen with 3DSBW. This was attributed to the yarn interlace in 3DSBW, where the warp yarns were placed at a larger angle, θ, to the thickness direction to hold the non-crimp weft yarns, than that in 3DSSW. So, the 3DSBW structure had heavier compactness, and in turn, imparted more friction between fiber and reed or heddle. However, the least fiber fracture was seen in 3DOW, due to the fact that the warp and weft yarns in 3DOW are non-crimp and are held together by the through-thickness z-yarns.

Based on the fiber fracture analysis among these three composites, the most serious fiber fracture was found in 3DSBW and was expected to have an adverse effect on the properties of the composite. 

### 3.3. Microstructure Analysis of Composites

It is reported that both fiber damage and yarn waviness also have significant negative effects on the properties of 3D woven composites [31,32]. Therefore, a microstructure analysis of the fiber orientation and crimping shall be beneficial. In this study, one micrograph of the cross-section was taken for each material type and at least six fibers (warp, weft and z-fiber) were measured in the different areas of the micrograph to gain the fiber waviness, as shown in Table S4.

**Figure 3:**
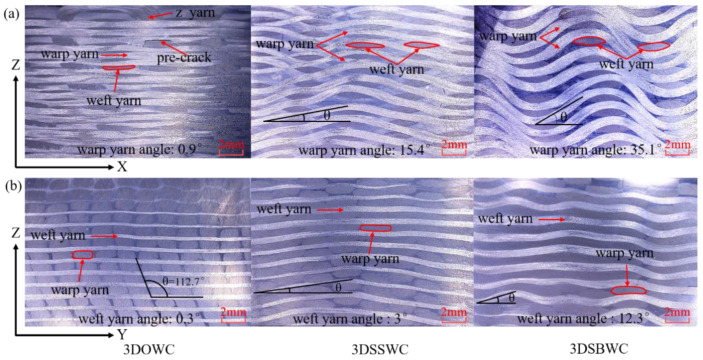
Cross-section of 3D composites along the (**a**) x-direction and (**b**) y-direction.

The cross-sections of 3D woven composites were shown in Figure 3. It can be seen that all three types of structures were distinctive for the warp yarns. For instance, the warp yarns were straight (warp waviness is 0.9 ± 0.2°) in the case of 3DOWC, in contrast, the warp yarns were seriously crimped to an angle of nearly 35.1 ± 4.5° for 3DSBWC. However, the warp yarn waviness in 3DSSW was in between 3DOWC and 3DSBWC (i.e., 15.4 ± 1.9°). The same observation could be made on weft yarns that the 3DSBW had the highest yarn inclination angle (i.e., 12.3 ± 1.3°), as shown in Figure 3b, due to the compaction between warp yarns and weft yarns.

For 3DOW structure, the z-yarns in thickness direction exhibited an “S” shape with the angle of inclination is 112.7 ± 1.6°, which was caused by the compression during the VARI process. Probably, this unfavorable effect is one of the causes of lower strength in the through-thickness direction due to significant fiber buckling.

### 3.4. Tensile Test

#### 3.4.1. In-Plane Tension (x and y-Direction)

**Figure 4:**
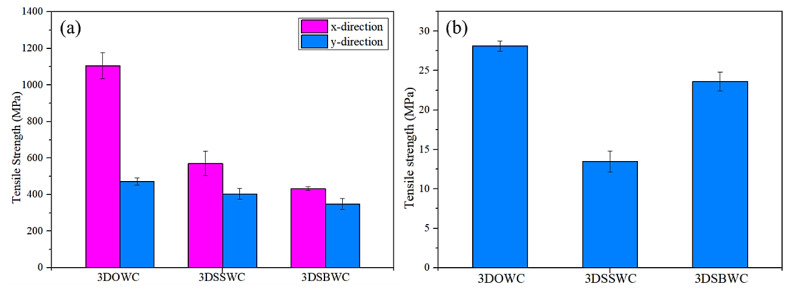
The tensile strength in (**a**) the x- and y-directions and (**b**) the z-direction.

The tensile strength of x and y-direction for all the samples were shown in Figure 4a. The largest gap in tensile strength was seen with 3DOWC. This is attributed to the yarns in 3DOWC being straight and the fiber content in the x-direction (i.e., warp density) being 4.5 times that of the y-direction (i.e., weft density), as shown earlier in Table 1. 

The tensile strength for 3DSBW and 3DSSW in the x-direction and y-direction were of much smaller differences, despite the fact that in both cases, as shown in Table 1 also, the fiber content in x-direction was about three times as much as in the y-direction. But considering the heavy warp yarn waviness and serious fiber damage in the x-direction and the straight weft yarns in the y-direction, it is not surprising that the x-directional tensile strength was compromised significantly. 

**Figure 5:**
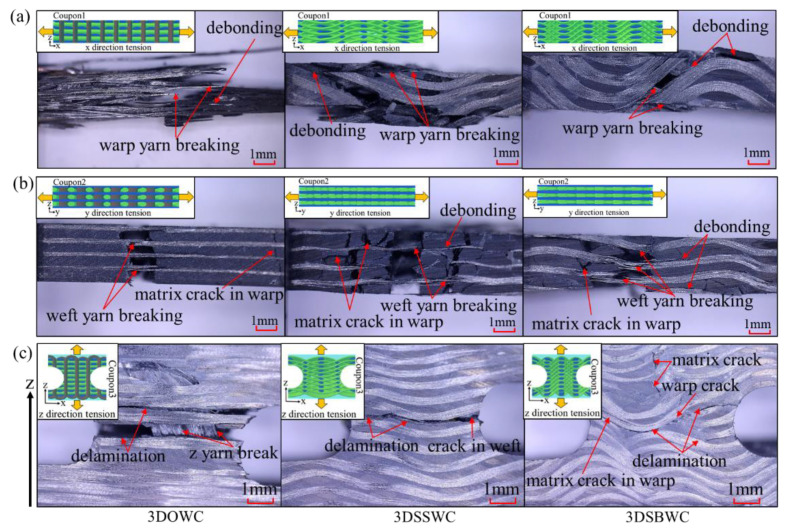
The tension failure mode in (**a**) the x-direction, (**b**) the y-direction, and (**c**) the z-direction.

In addition, the tensile strengths in the same direction were significantly different for these three structures, even when the fiber content in that direction was kept the same. This is attributed to the different yarn waviness and the fiber fracture. The x-directional tension failure of 3DOWC hairbrush, as often observed in cases of unidirectional composite tensile tests, usually yields a high strength, and is the most desirable tensile failure mode, as shown in Figure 5a Therefore, the highest rate of force growth and modulus were seen in 3DOWC due to the straight yarn (Figure S6a,c). 

Contrarily, the significant fiber crimp in the x-direction of 3DSSWC and 3DSBWC led to severe stress concentration under tension that resulted in debonding and yarn breakage (see Figure 5a), and consequently, much lower tensile strengths than the fiber would have shown without any waviness and less fiber fracture. Furthermore, the force in 3DSSWC and 3DSBWC grew slowly because the crimp yarns were stretching during the test and resulted in the 3DSSWC and 3DSBWC having a lower tensile modulus than 3DOWC (Figure S6a,c).

The yarns in the y-direction of 3DOWC were straight mostly and had less fiber fracture, as shown in Figure 5b and Figure 2, yielding the highest tensile strength amongst the group. The coupons of 3DSBWC exhibited apparent matrix cracking owing to its fiber crimp, and therefore limiting its weft fiber ability to carry loads. Consequently, it had the lowest y-directional tensile strength within the group. However, the force growth trend and tensile modulus (Figure S6b,d) were similar because little difference in the yarn crimps in this direction was seen in these composites. 

The above-shown failure modes agreed well with the obtained in-plane tensile strengths, confirming that the 3DOWC had a clear in-plane advantage, which was controlled by the yarn waviness and fiber fracture in the test direction. 

#### 3.4.2. Out-Plane Tension (z-Direction)

The tensile strengths and failure modes of all the three samples in through-thickness/out-plane (z-direction) was shown in Figure 4b and Figure 5c. 3DOWC had the highest z-directional tensile strength owing to the presence of z-yarns in the weave styles. The z-yarns breakage failure mode also provided a clear picture that the z-yarns in the 3DOWC provided direct load-bearing, which yielded the highest tensile strength among these samples (see Figure 5c).

It can be established that because of the lacking of z-yarns in 3DSSWC and 3DSBWC, the crimped warp yarns provided additional load-bearing in the z-direction. Nonetheless, the crimping results in stress concentration, thus causing the cracking in the interlacing area of warp and weft yarns, and then propagating along the warp yarn direction, as shown in Figure 5c. In general, the heavier the warp yarn crimping, the more energy was required for the crack to expand along the warp direction, which can be seen in Figure S7; the force was dropped stage-by-stage instead of sharply dropping. Therefore, the z-directional tensile strength of 3DSBWC was higher than that of 3DSSWC. Moreover, the 3DSSW cross-section resembled closely that of a 2D laminate, and hence it showed a low z-directional tensile strength. 

The results in this section confirmed that the 3DOW style offered the highest overall tensile strength compared to the 3DSSW and 3DSBW structures when combining both in-plane and out-plane directions. In addition, the yarn waviness has a negative effect on the in-plane tensile strength and modulus, but a positive effect on the z-direction strength and modulus (Table S5).

### 3.5. Compression Test

#### 3.5.1. In-Plane Compression (x and y-Direction)

**Figure 6:**
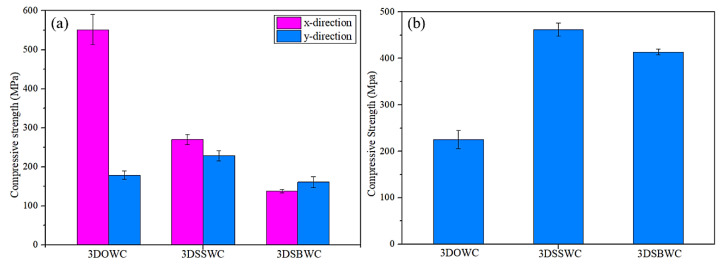
The compressive strength of (**a**) x-direction and y-direction and (**b**) z-direction.

**Figure 7:**
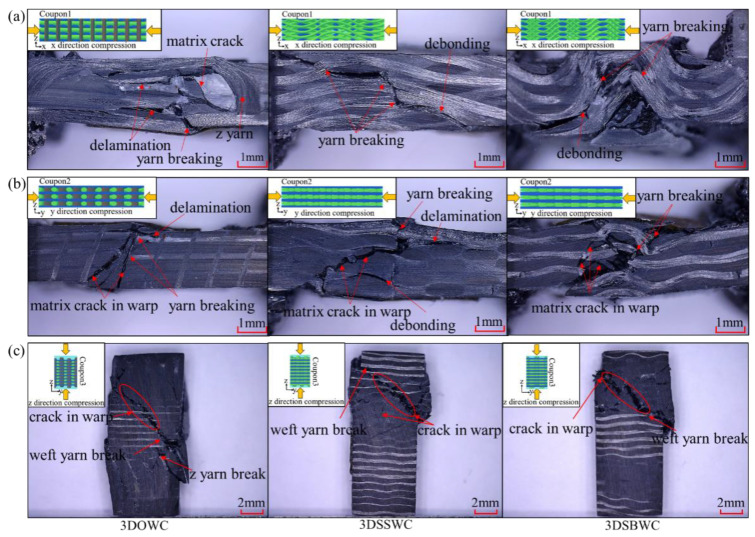
Compression failure mode of 3D composites in (**a**) the x-direction, (**b**) the y-direction, and (**c**) the z-direction.

The in-plane compression strengths and failure mode of the three composites were shown in Figure 6a and Figure 7a,b. It can be seen that the x-directional compression failure of 3DOWC mainly appeared in the area lacking z-yarns with the warp tow and matrix crack and the delamination between warp yarns. Thus, the 3DOWC with the straight warp yarn was responsible for the highest x-directional compressive strength. However, the significant fiber buckling in the x-direction of 3DSSWC and 3DSBWC led to the failure mode in 3DSSWC, and 3DSBWC was dominated by debonding and fiber breakage, as shown in Figure 7a, and had much lower compressive strength than the fiber would have shown if without any waviness.

In the y-direction, as shown in Figure 7b, the crack angles of the three composites were different, which was attributed to the yarn crimping. The fiber buckling in 3DSBWC composite triggered apparent matrix cracking, and restricted y-directional yarns to bear the compressive load. Thus, 3DSBWC showed the lowest compressive strength (Figure 6a). 

In addition, the compressive strengths of x- and y-directions were not the same, which was attributed to the combining effect of fiber density, yarn waviness, and weaving damage in the corresponding direction. For example, the 3DSBWC with the large warp yarns waviness and serious fiber damage in the x-direction and the straight weft yarns in the y-direction, resulting in the difference of compressive strengths in the x- and y-direction, was much smaller, even though the discrepancy of fiber content of 3DSSWC and 3DSBWC in x- and y-direction was also higher. 

The effects of yarn waviness included not only on the compressive strength but also the mechanical behavior and modulus, as shown in Figure S8 and Table S6. In x-direction, the 3DOWC with straight yarn had the highest compressive modulus and linear load-displacement curve which dropped sharply. However, the 3DSBWC was not sharply dropped due to crimped yarn in its structure and thus possesses the lowest compressive modulus (Figure S8a,c). The moduli (Figure S8b,d) were basically the same due to little difference in the yarn crimp in y-direction.

The above failure mode analysis and mechanical behavior agree well with the in-plane compressive strength test data, which validated that yarn alignment and minor waviness in the test direction of 3DOWC were responsible for its high strength over the other two weaving styles of reinforced composites. 

#### 3.5.2. Out-Plane Compression (z-Direction)

The z-directional compressive strengths behaved quite differently compared to that of the z-directional tensile strengths. As shown in Figure 6a, it is interesting to see the ranking of compressive strength in the z-direction (i.e., 3DSSWC > 3DSBWC > 3DOWC) corresponds to the weft yarn density (y-direction), as shown earlier in Table 1. But considering the fact that the weft yarn failure mode was shear failure (Figure 7c) and the modulus obtained from the stress-strain curve (Figure S9b) was nearly the same, it is not surprising that the main load carrier was the weft yarn among these three composites and the 3DSSWC with the highest fiber content (i.e., weft density: 3.4 picks/cm) had the highest out-plane compressive strength. 

For 3DSSWC and 3DSBWC under z-directional compression, the stress concentration first occurred in the interlacing area of warp and weft yarns and then transferred to the warp and weft yarns, where cracks would be expected. However, the high warp fiber content and warp yarn waviness forced the cracking to move to weft yarn breakage, because of its low weft fiber content, as shown in Figure 7c. Therefore, the trends of their load-displacement curves were similar, as shown in Figure S9a.

Although there was the presence of z-yarns in 3DOWC to serve as the load-bearing in the through-thickness direction, its z-directional compressive strength was much lower than 3DSSWC and 3DSBWC. This is due to the fact that there were only 4.5 z-yarns in each test coupon and even more, unfortunately, these z-yarns were not aligned to the test direction due to the compression from the vacuum bag during composite fabrication. The failure mode of the 3DOWC and the load-displacement curve of 3DOWC (Figure S9a) provided evidence to support this observation. It can be seen that in 3DOWC compressive coupons, the crack was apparently expanded along the z-yarn due to shear failure, which means that the inclined z-yarns could hardly prevent the crack propagation, resulting in the lowest z-directional compressive strength among three composites.

In conclusion, the results in this section demonstrated that, when considering the in-plane direction, the 3DOW style offered the highest x-directional compressive strength. But the highest y-directional and z-directional compressive strength was provided by 3DSSW structure due to it having the highest fiber content along the test directions. Likewise, the yarn crimp also has a negative effect on the in-plane compressive strength and modulus, but the effect on the z-direction was related to the failure mode.

### 3.6. Flexural Test

#### 3.6.1. In-Plane Flexural Strength

**Figure 8:**
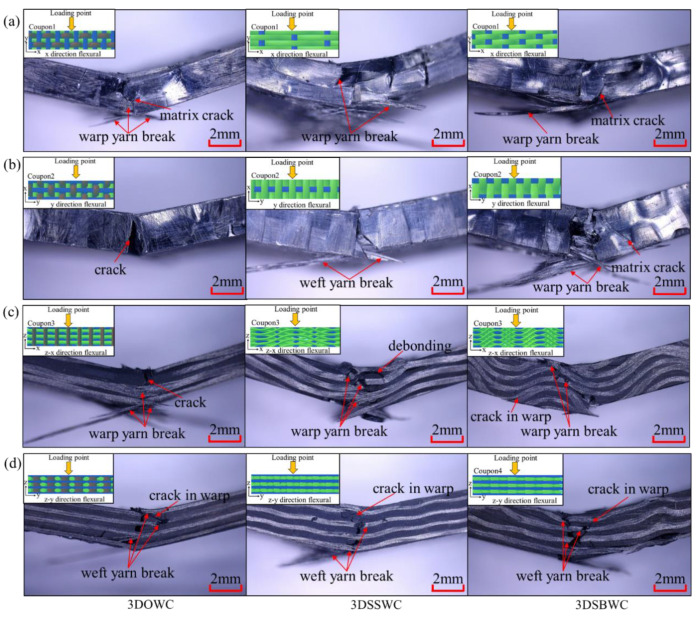
Flexural failure mode in (**a**) x-direction, (**b**) y-direction, (**c**) z-x direction, and (**d**) z-y direction.

**Figure 9:**
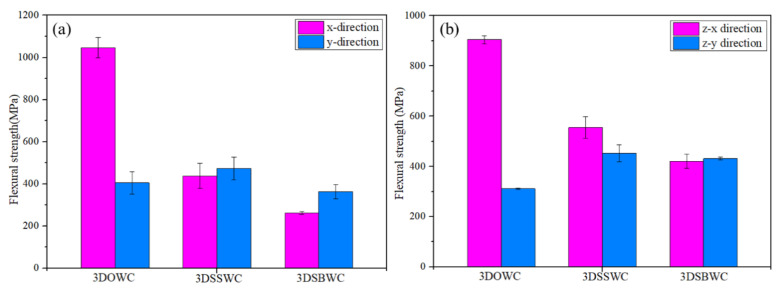
The flexural strength of (**a**) x- and y-direction and (**b**) z-x and z-y directions.

The correct failure mode is critical in flexural strength measurement. In both x- and y-directions, the failure modes were all tensile with fiber breaking amongst three composites, as shown in Figure 8a,b, which was the correct failure mode. Therefore, the flexural strength in x- and y-directions was decided by the state of the warp and weft yarns, respectively. Thus, the 3DOWC with straight warp yarns and few fiber damages were responsible for its highest flexural strength in x-direction (Figure 9a). The 3DSSW with the highest fiber content in the y-direction (i.e., weft density 3.4 picks/cm) combined with the straight weft yarns, compared to the other two types of woven structures, showed in Figure 9a, led to its highest y-directional flexural strength. 

Moreover, due to the same failure mode in x and y-direction, the difference of the flexural strength between x and y-direction was mainly affected by the fiber content and fiber status (i.e., fiber bulking and damage) in the test direction. Therefore, it was not surprising that the largest discrepancy between x and y-direction was once again seen with 3DOWC. The yarn’s waviness also had a negative effect on the mechanical behavior and the modulus, resulting in the 3DSBWC having a gradual failure process and lowest flexural modulus, as shown in Figure S10a,c. However, the similar failure process and modulus were also seen in Figure S10b,d due to the similar weft yarn waviness.

In conclusion, combining the in-plane flexural strength and modulus listed in Table S7 and flexural failure mode analysis, the 3DOWC had a clear in-plane flexural strength and modulus advantage because of its straight yarns in the x-direction.

#### 3.6.2. Out-Plane Flexural 

Considering the different yarn patterns in z-x directional and z-y directional flexural test coupons, as shown in Figure S11, it is necessary to divide the out-plane flexural test into z-x and z-y directions. 

Figure 8c,d indicated that the failure modes in z-x and z-y directions were all tension failures with fiber breakage. It means that that flexural strength was controlled by the yarns in the corresponding direction. Therefore, the 3DOWC with straight warp yarns and few fibers damage had the highest z-x flexural strength amongst this group, and the 3DSSWC had the highest z-y directional flexural strength due to its straight weft yarns and high fiber content in this direction, as shown in Figure 9b.

Furthermore, the different failure process and flexural modulus shown in Figure S12a,c relate to the yarn waviness in the testing direction. For example, the high warp yarn crimp in 3DSBWC resulted in the force being gradually decreased and the lowest flexural modulus (Table S7) among these composites. However, the failure procedure and modulus shown in Figure S12b,d were almost similar due to the straight weft yarn in z-y direction.

Evidently, the flexural strengths between z-x and z-y directions were different as shown in Figure 9b. The difference was most significant with 3DOWC, but the flexural strengths in the z-x and z-y directions for both 3DSSWC and 3DSBWC were more or less the same. The gained results are due to the combined effect of different yarn waviness and fiber content between z-x and z-y direction. 

The above-shown failure modes agreed well with the observed in-plane and out-plane flexural strength, confirming that the flexural strength was again controlled by the yarn waviness and fiber content in the test direction. Therefore, the flexural strength of 3DOWC was significantly better than the 3DSSWC and 3DSBWC in both x and z-x directions, while the flexural strength in the y and z-y directions was relatively close.

## 4. Conclusions 

Under the same weaving parameters, the 3DOW woven style can provide a clear advantage to tensile and flexural strengths in all three directions when compared with 3DSSW and 3DSBW structures. This is attributed to the 3DOW having straight yarn and slight fiber damage during the weaving process. For the compressive strength, the only shortage for the 3DOW woven style is that the 3DOW has the lowest z-direction compressive strength due to the low content of z-yarns and yarns inclination in the z-direction. For 3DSSW and 3DSBW, the in-plane strength was seriously decreased due to the yarn waviness and fiber damage, which will enable stress concentration during the whole process. Therefore, from this study, in the choice of 3D reinforced structures for mix or complex loading application, 3DOW is the most suitable structure.

## Figures and Tables

**Table 1:** The specifications of 3D woven fabric.

Structures	Thickness (mm)	Warp Density (Pick/cm)	Weft Density (Pick/cm)	Layers (Warp/Weft)	Fiber Volume Fraction (%)
3DOW	16.5	9.0	2.0	21/22	44.3
3DSSW	17.0	9.0	3.4	21/21	45.5
3DSBW	16.0	9.0	3.3	21/22	47.7

## Supplementary Materials

The following are available online at https://www.mdpi.com/1996-1944/13/12/2765/s1, Figure S1: Structures of the 3D woven fabric: (a) 3DOW (b) 3DSSW (c) 3DSBW, Figure S2: Illustration of VARI process lay-up, Figure S3: Coupon definition and dimension for the tensile test at (a) x-, y- and (b) z-directions, Figure S4: Coupon definition and dimensions for compression test at (a) x-, y- and (b) z-directions, Figure S5: Coupon definition and dimension for flexural test at (a) x-, y- and (b) z-x, z-y directions, Figure S6: Quasi static nominal load–displacement curve for 3D woven composites under tensile test, in both (a) warp/x and (b) weft/y directions. Normal stress vs. normal strain for tensile test in (c) warp/x and (d) weft/y directions, Figure S7: (a) Quasi static nominal load–displacement curve for 3D woven composites in the z- direction tensile test. (b) Normal stress vs. normal strain for tensile test in z direction, Figure S8: Quasi static nominal load–displacement curve for 3D woven composites under compressive test, in both (a) warp/x and (b) weft/y directions. Normal stress vs. normal strain for compressive test in (c) warp/x and (d) weft/y directions, Figure S9: (a) Quasi static nominal load–displacement curve for 3D woven composites in the z- direction compressive test. (b) Normal stress vs. normal strain for compressive test in z direction, Figure S10: Quasi static nominal load–displacement curve for 3D woven composites under flexural test, in both (a) warp/x and (b) weft/y directions. Normal stress vs. normal strain for flexural test in (c) warp/x and (d) weft/y directions, Figure S11: Illustration of yarn pattern in (a) the z-x directional coupon and (b) the z-y directional coupon, Figure S12: Quasi static nominal load–displacement curve for 3D woven composites under flexural test, in both (a) the z-x and (b) z-y directions. Normal stress vs. normal strain for flexural test in (c) z-x and (d) z-y directions, Table S1: The properties of T800 carbon fiber, Table S2: The properties of epoxy resin BAC172, Table S3: The thickness of unit cell in 3D woven composite, Table S4: The measurement data for fiber waviness, Table S5: Engineering constants obtained for in-plane loading and out-plane tension, Table S6: Engineering constants obtained for in-plane loading and out-plane compression, Table S7: Engineering constants obtained for in-plane loading and out-plane flexural.
