# Supplementary material for: A Comprehensive Study on the Mechanical Properties of Different 3D Woven Carbon Fiber-Epoxy Composites
Source: Materials (Basel). 2020 Jun 18;13(12):2765. doi: 10.3390/ma13122765 (PMC7344398; doi:10.3390/ma13122765)
Supplement: Supplementary file 1 [file materials-13-02765-s001.pdf]

A Comprehensive Study on the Mechanical Properties of Different 3D Woven Carbon Fiber-Epoxy Composites

Qiaole Hu [1,2], Hafeezullah Memon [2,3], Yiping Qiu [2,3,4], Wanshuang Liu [2,3,*] and Yi Wei [2,3,*]

[1] School of Textile and Garment, Anhui Polytechnic University, Wuhu, Anhui 241000, China; huqiaole@ahpu.edu.cn
[2] Key Laboratory of Textile Science &Technology, Ministry of Education, College of Textiles, Donghua University, 2999 North Renmin Road, Shanghai 201620, China; hm@mail.dhu.edu.cn (H.M.); ypqiu@dhu.edu.cn (Y.Q.)
[3] Donghua University Center for Civil Aviation Composites, Donghua University, 2999 North Renmin Road, Shanghai 201620, China
[4] College of Textiles and Apparel, Quanzhou Normal University, Quanzhou, Fujian 362000, China
* Correspondence: wsliu@dhu.edu.cn (W.L.); weiy@dhu.edu.cn (Y.W.)

Received: 19 May 2020; Accepted: 11 June 2020; Published: date

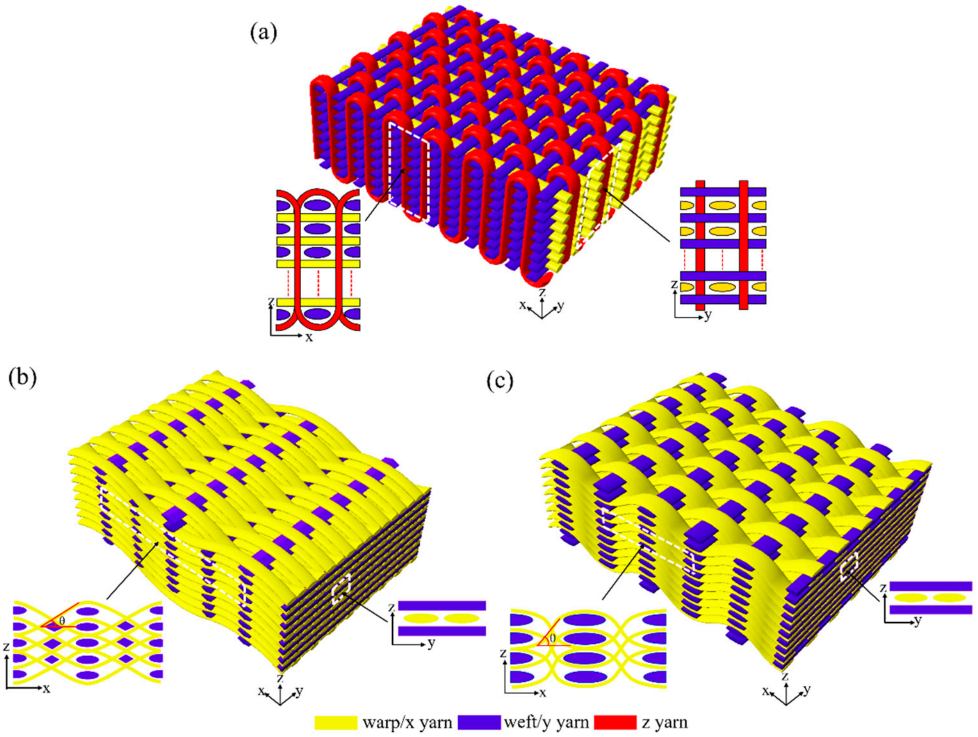

Figure S1. Structures of the 3D woven fabric: (**a**) 3DOW (**b**) 3DSSW (**c**) 3DSBW.

As shown in Figure S1a, the 3DOW fabric was woven with an orthogonal fiber structure, consisting in-plane non-crimp warp (i.e., yellow color) and weft yarns (i.e., blue color) interlaced in the through-thickness direction with the z-binder yarns (i.e., red color). While, for the 3DSSW and 3DSBW, only the warp (i.e., yellow color) and weft yarns (i.e., blue color) were contained, but the warp yarns were placed at an angle, θ, to the thickness direction to hold the non-crimp weft yarns together, and the angle in 3DSBW was greater than 3DSSW, as shown in Figure S1b,c.

Table S1. The properties of T800 carbon fiber.

Type	Yarn Density g/km	Tensile Strength (MPa)	Tensile Modulus (GPa)	Elongation At Break (%)	Sizing Content (%)
T800 SYT55S-12K	447–452	5890–6222	290–292	2.0–2.1	1.35–1.43

Table S2. The properties of epoxy resin BAC172.

Type	Density (g/cm3)	Tensile Strength (MPa)	Tensile Modulus (MPa)	Tensile Elongation %	Flexural Strength (MPa)	Flexural Modulus (MPa)
BAC172	1.2	75	2850	7.6	130	3500

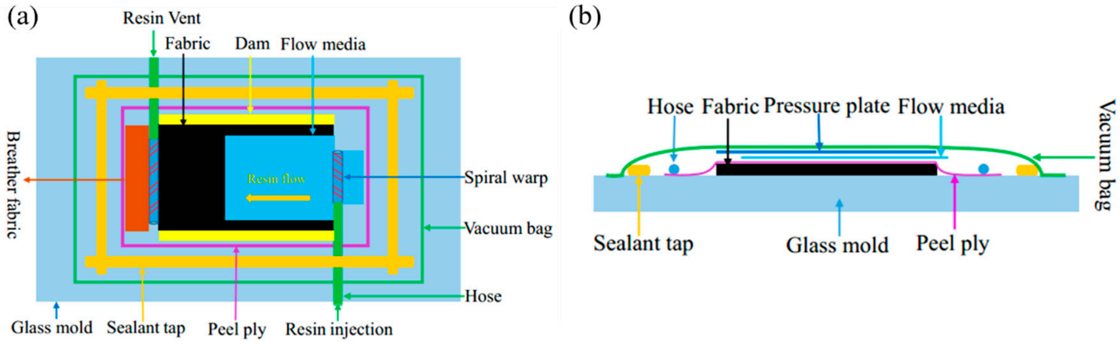

Figure S2. Illustration of VARI process lay-up.

In this process, the lay-up procedure was shown in Figure S2b and two thickness gauges (i.e., dam and glass pressure plate, shown in Figure S2a,b) were used to keep the thickness and fiber volume consistent.

Table S3. The thickness of unit cell in 3D woven composite

t_{unit} (um)	1	2	3	4	5	6	Average	Standard Deviation
3DSSWC	632.9	626.8	680.7	627.8	657.3	631.9	642.9	21.6
3DSBWC	1464	1496	1904	1432	1478	1564	1556	175.9

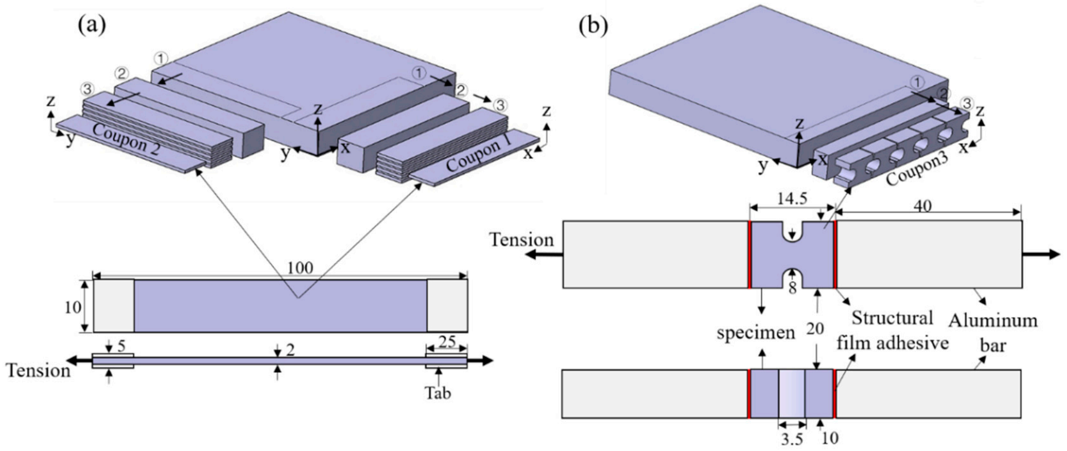

Figure S3. Coupon definition and dimension for the tensile test at (**a**) x-, y- and (**b**) z-directions.

The tension test coupon cutting and dimensions are shown in Figure S3. First, the composites were cut along the x- or y-direction, and the coupons with 14.5 mm were gained, as shown in step ②. Secondly, this coupon was sliced in the direction perpendicular to z-direction to obtain the x- or y-direction coupons, as shown in step ③. Therefore, the x- or y-direction coupons were machined. While, the z-direction coupons were obtained by the first cut along the x-direction, as shown in stage ②, and then cut in the direction parallel to the z-direction, as shown in ③. In order to provide adequate support during the test, two aluminum blocks were subsequently bonded to the top and bottom surfaces of the coupon using structural film adhesive, as shown in Figure S3a. In this figure, Coupon 1 was for x-direction test and Coupon 2 was for y-direction test.

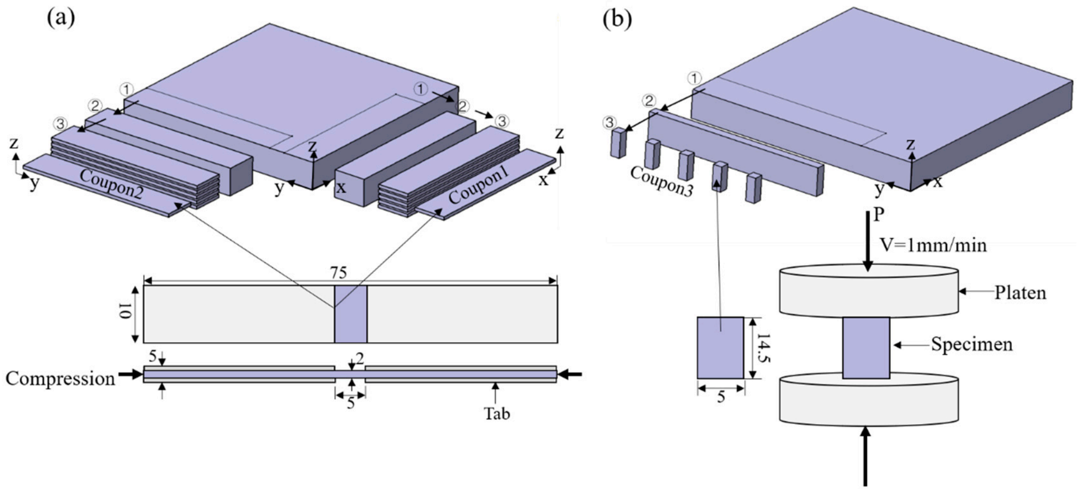

Figure S4. Coupon definition and dimensions for compression test at (**a**) x-, y- and (**b**) z-directions.

The cutting procedure for compression coupon preparation is the same as the tensile test and dimensions of test coupon are shown in Figure S4. For the compressive coupons at x/warp and y/weft directions, stiffener tabs made of glass fiber laminates were used and the gauge length was 5 mm (Figure S4a). The compression coupons cut out at z-direction was used for the test without tabs (Figure S4b).

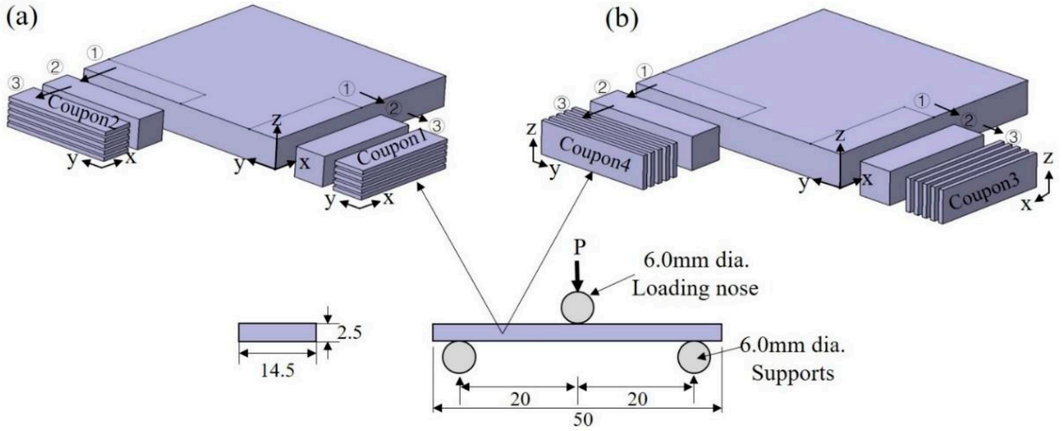

Figure S5. Coupon definition and dimension for flexural test at (**a**) x-, y- and (**b**) z-x, z-y directions.

In the Figure S5a, Coupon 1 and Coupon 2 are for the flexural tests at x/warp and y/weft directions, respectively. Two types of coupons (Coupons 3 and 4) were prepared for the test at z-x and z-y directions, respectively (Figure S5b).

Table S4. The measurement data for fiber waviness.

	Fiber Waviness	1	2	3	4	5	6	Average	St. Dev
3DSSWC	Warp	13.4	17.5	14.9	16.1	13.0	17.5	15.4	1.94
	Weft	3.3	2.9	2.5	3.3	3.0	3.3	3.0	0.33
3DSBWC	Warp	40.8	32.9	29.1	38.2	37.7	31.6	35.1	4.52
	Weft	12.3	13.7	13.6	11.5	10.4	12.1	12.3	1.3
3DOWC	Warp	0.7	1.1	1.1	1.0	0.9	0.8	0.9	0.2
	Weft	0.4	0.3	0.3	0.4	0.3	0.4	0.3	0.04
	z-Fiber	64.4	67.3	68.3	66.8	67.7	69.1	67.3	1.63

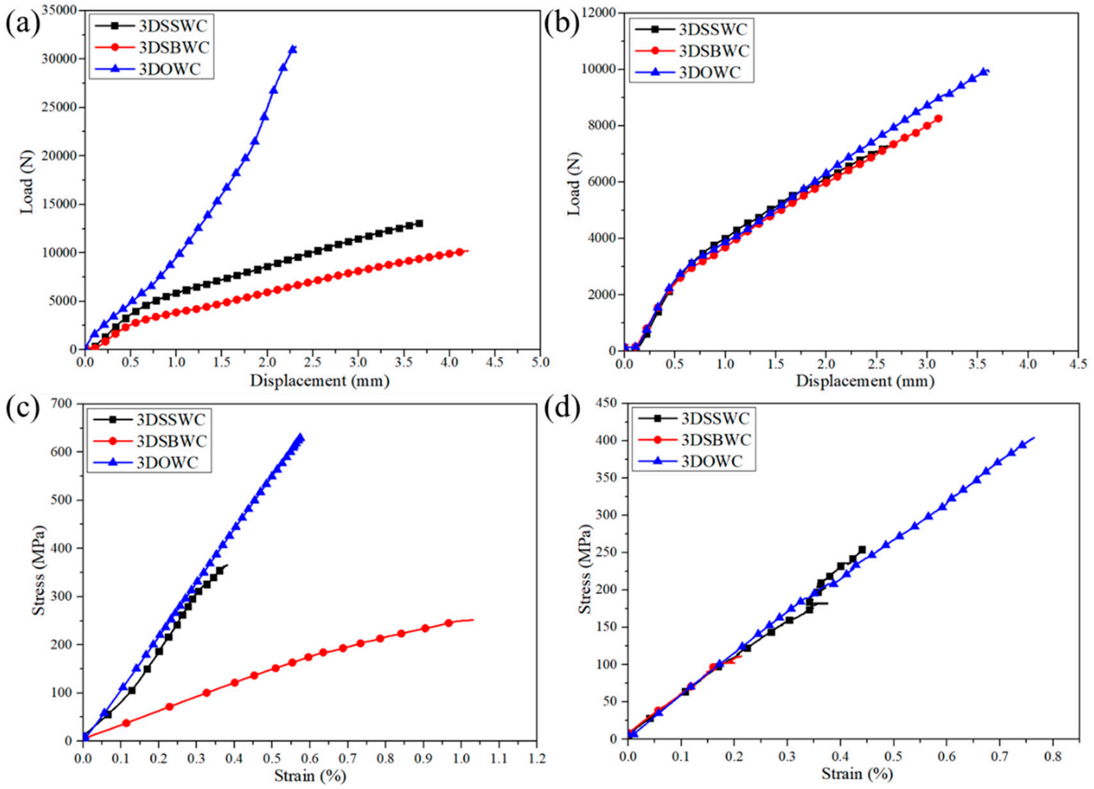

Figure S6. Quasi static nominal load–displacement curve for 3D woven composites under tensile test, in both (**a**) warp/x and (**b**) weft/y directions. Normal stress vs. normal strain for tensile test in (**c**) warp/x and (**d**) weft/y directions.

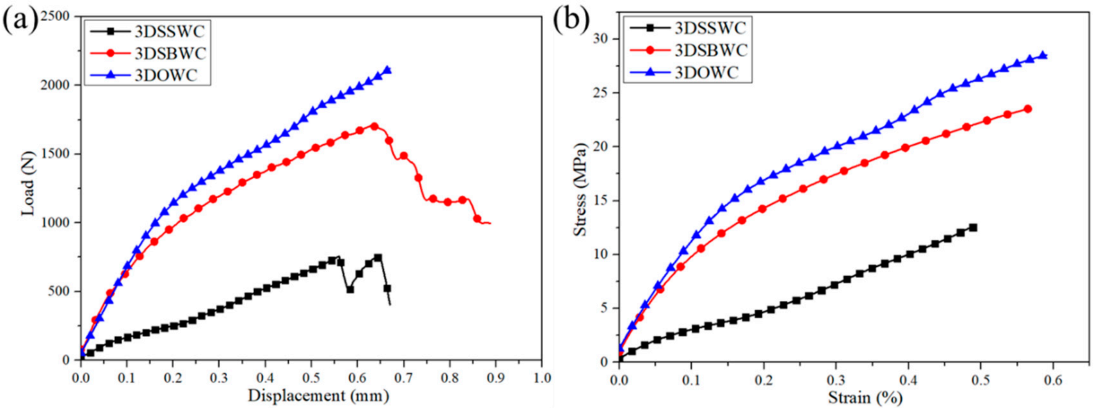

Figure S7. (**a**) Quasi static nominal load–displacement curve for 3D woven composites in the z-direction tensile test. (**b**) Normal stress vs. normal strain for tensile test in z direction.

Table S5. Engineering constants obtained for in-plane loading and out-plane tension.

Structure	T_{warp} (MPa)	M_{warp} (GPa)	T_{weft} (MPa)	M_{weft} (GPa)	T_z (MPa)	M_z (GPa)
3DSSWC	570	78	404	50	13	2.7
3DSBWC	432	29	349	49	24	8.6
3DOWC	1105	131	471	51	29	9.8

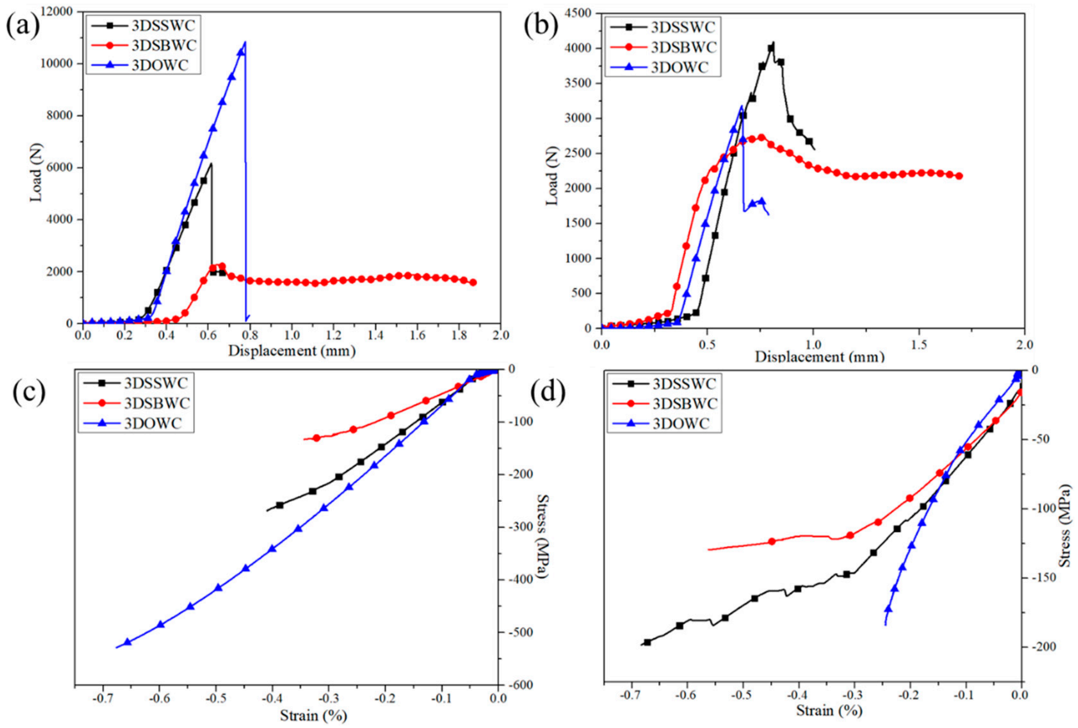

Figure S8. Quasi static nominal load–displacement curve for 3D woven composites under compressive test, in both (**a**) warp/x and (**b**) weft/y directions. Normal stress vs. normal strain for compressive test in (**c**) warp/x and (**d**) weft/y directions.

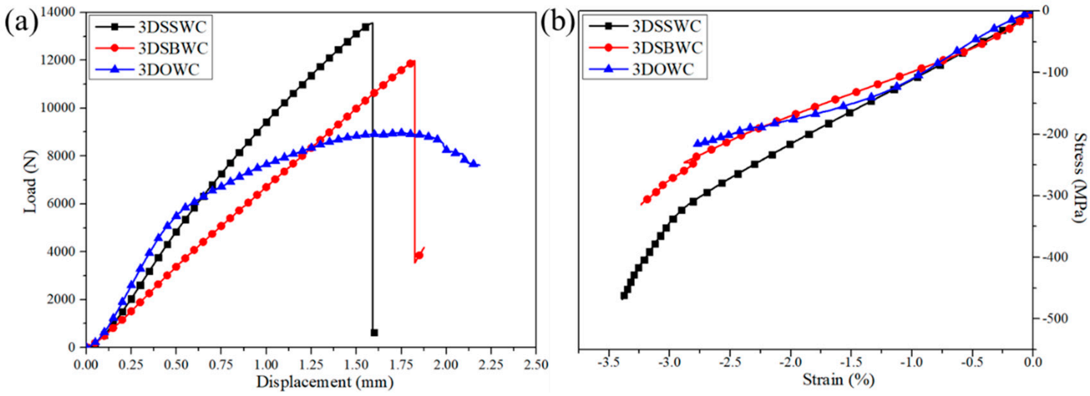

Figure S9. (**a**) Quasi static nominal load–displacement curve for 3D woven composites in the z-direction compressive test. (**b**) Normal stress vs. normal strain for compressive test in z direction.

Table S6. Engineering constants obtained for in-plane loading and out-plane compression.

Structure	C_{warp} (MPa)	M_{warp} (GPa)	C_{weft} (MPa)	M_{weft} (GPa)	C_{z} (MPa)	M_{z} (GPa)
3DSSWC	270	78	229	45	462	11
3DSBWC	138	51	161	40	413	12
3DOWC	474	83	178	41	225	12

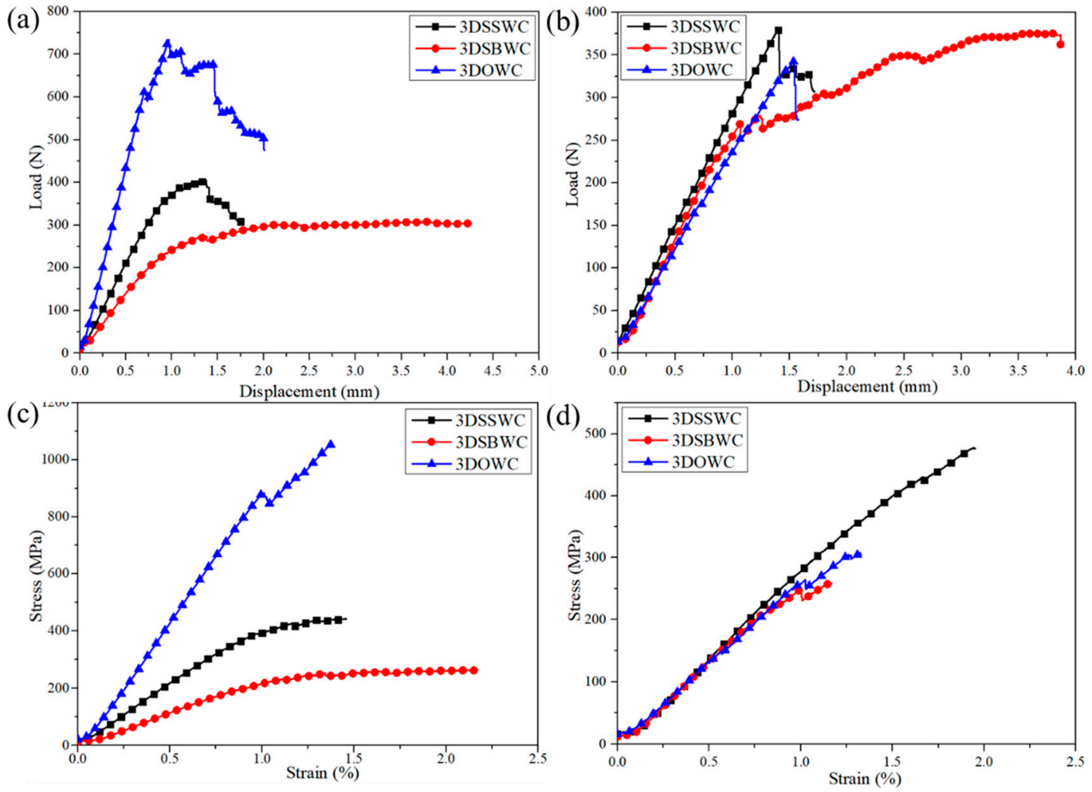

Figure S10. Quasi static nominal load–displacement curve for 3D woven composites under flexural test, in both (**a**) warp/x and (**b**) weft/y directions. Normal stress vs. normal strain for flexural test in (**c**) warp/x and (**d**) weft/y directions.

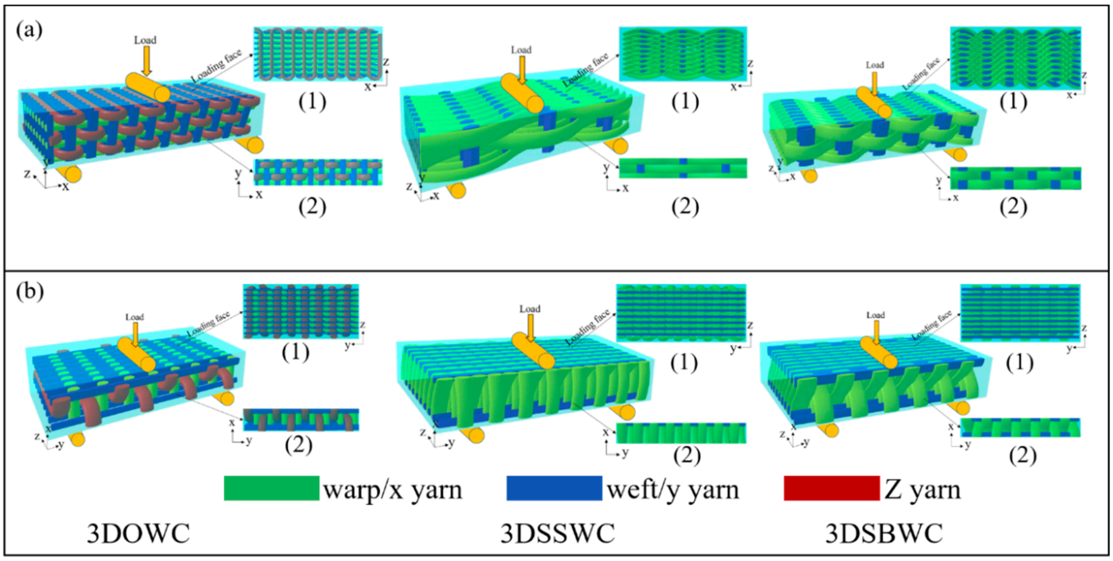

Figure S11. Illustration of yarn pattern in (**a**) the z-x directional coupon and (**b**)the z-y directional coupon.

It is important to note that, although the yarn layers in the z-x and z-y directional test coupons were the same (i.e., 21 layers), the fiber content in these test coupons, as shown in Figure S11, was different, which was decided by the yarn density (i.e., z-x direction: warp density 9 picks/m, z-y direction: 3DOW 2 ends/cm, 3DSSW 3.4 ends/cm, 3DSBW 3.3 ends/cm).

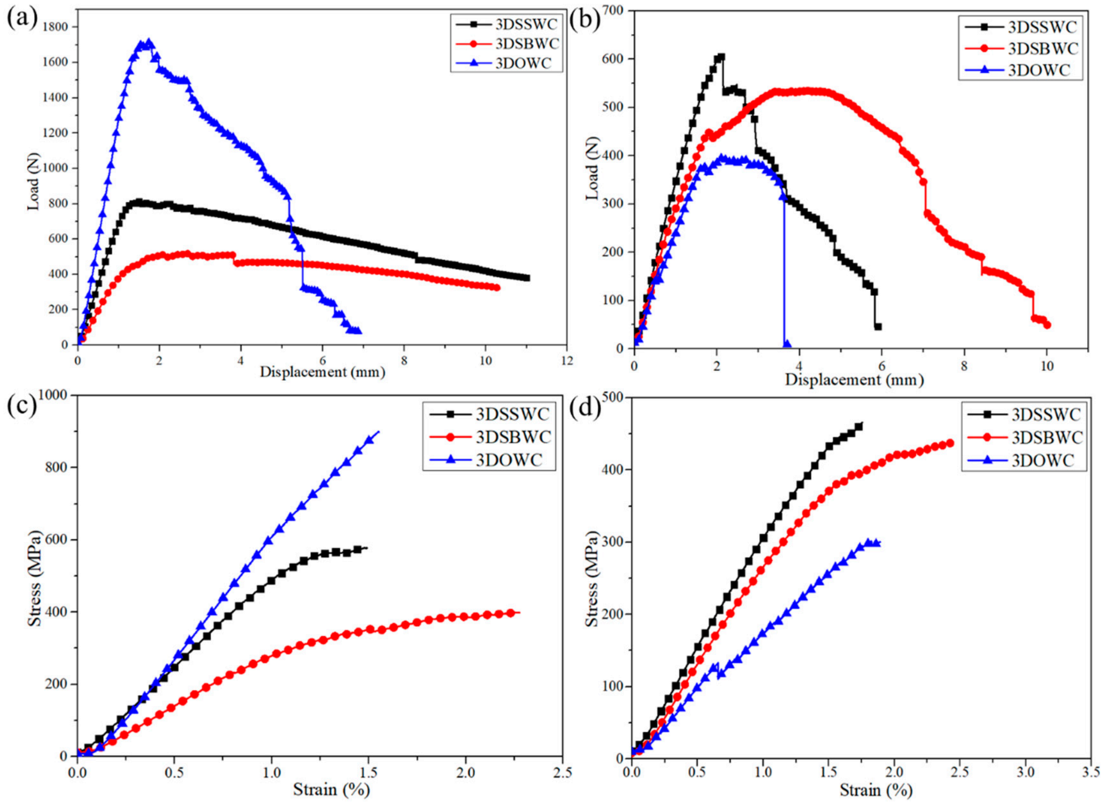

Figure S12. Quasi static nominal load–displacement curve for 3D woven composites under flexural test, in both (**a**) the z-x and (**b**) z-y directions. Normal stress vs. normal strain for flexural test in (**c**) z-x and (**d**) z-y directions.

Table S7. Engineering constants obtained for in-plane loading and out-plane flexural.

Structure	F_{warp} (MPa)	M_{warp} (GPa)	F_{weft} (MPa)	M_{weft} (GPa)	$F_{Z\text{-}warp}$ (MPa)	$M_{Z\text{-}warp}$ (GPa)	$F_{Z\text{-}weft}$ (MPa)	$M_{Z\text{-}weft}$ (GPa)
3DSSWC	262	40	477	34	555	51	452	29
3DSBWC	439	24	363	31	421	31	431	27
3DOWC	1046	89	405	26	905	67	312	22

© 2020 by the authors. Submitted for possible open access publication under the terms and conditions of the Creative Commons Attribution (CC BY) license (http://creativecommons.org/licenses/by/4.0/).
